# Accurate radiographic interpretation of misfit milled zirconia crowns of different designs: An *in vitro* study

**DOI:** 10.1371/journal.pone.0338690

**Published:** 2026-01-08

**Authors:** Mohammad Barayan, Khadijah M. Baik

**Affiliations:** 1 Oral and Maxillofacial Radiology, Oral Diagnostic Science Department, Faculty of Dentistry, King Abdulaziz University, Jeddah, Saudi Arabia; 2 Oral and Maxillofacial Prosthodontics Department, Faculty of Dentistry, King Abdulaziz University, Jeddah, Saudi Arabia; Kuwait University, Faculty of Dentistry, KUWAIT

## Abstract

Prosthodontists often assess crown margins clinically and radiographically. However, judging an open crown margin clinically is difficult, and their radiographic assessment is controversial. The objective of this study was to investigate factors influencing interpretation of crown margins to optimize their assessment. Nine plastic KaVo model teeth were divided into three groups (n = 3 per group), and each group received a different margin design (slanted buccal or lingual, concave coronal or convex, 0.7 mm or 1 mm finish line). Zirconia crowns were fabricated, and preparations were exposed to five different radiographic angulations: 1) perpendicular to both the plate and teeth; 2) with 10° of mesial shift; 3) with 10° of distal shift; 4) with +10° vertical angulation; and 5) with −10° vertical angulation. Images were interpreted for open/closed margins by three consultants. Binary logistic regression was used to determine predictors of an open margin, and intra- and inter-examiner reliability was assessed. Overall, closed margins (false negatives) were most detected when the PID was at +10° vertical angulation (72.2% of the time), independent of margin design. Predictors of an open crown margin were position-indicating device (PID) angles at right angles to the tooth and 10° mesial shift when the margin was 0.7 mm. However, it was less likely that a slanted buccal open crown margin would be detected with +10° vertical angulation. The radiographic interpretation of the margins of zirconia crowns is significantly influenced by PID angulation and margin design. A + 10° vertical angulation should be avoided when assessing marginal adaptation, and a convex coronal finish line of <1 mm should be avoided as they are harder to detect radiographically when open.

## Introduction

The long-term survival of indirect extra-coronal restorations relies on close margin adaptation [1]. Clinically, a small margin gap (40–120 microns) is acceptable [2–4], but larger gaps place the restoration at risk of cement wash-out by saliva followed by bacterial ingress, gingival inflammation, and recurrent caries [5]. Evaluating crown margin fit prior to final cementation is one of the most important steps in fixed partial prosthesis fabrication and success, as cementation of a prosthesis with an open margin can lead to early failure. Some clinical scenarios require extension of the finish line subgingivally, and in these cases assessment of crown margin adaptation can be challenging. In practice, dentists may accept larger gaps when crown margins are placed subgingivally [6], and such gaps may allow luting agents to contact the periodontium, leading to gingival inflammation and progressive periodontal disease [7].

Crown margins can be evaluated by tactile sensation using an explorer, visual inspection, or radiographic evaluation, and each method has its limitations. Tactile sensation is subjective and may not be consistent between dentists, with detection of subgingival and supragingival margins using an explorer producing very different results (34–119 microns subgingivally vs 2–51 microns supragingivally) [6]. Subgingival margins can be impossible to visually inspect, while radiographs can fail to detect small crown openings [8]. Therefore, combined visual inspection and radiography is recommended for subgingival margins [9,10], while supragingival margins can be evaluated using an explorer and visualization [6].

In the laboratory, two protocols can be used to evaluate the marginal fit of a crown: the non-invasive replica technique [11–13] and the invasive cross-sectional technique [14]. In the non-invasive replica technique, the crown is filled with a light body polyvinylsiloxane impression material and seated on the die. Following setting of the material, the crown is removed, and a heavy body impression material is injected on top of the light body for stabilization. After the heavy body has set, the replica is sectioned using a scalpel and the gap between the die and the crown is measured by microscopy at 50x magnification. In the invasive cross-sectional technique, the crown is cemented on the die and, 24 hours after cementation, the crown is then embedded in gypsum and allowed to set. Following setting, the crown is sectioned from mesial to distal and from buccal to lingual and inspected by microscopy at 50x magnification. While comparative studies of the two techniques show equivalence for measuring the crown marginal fit [15], the non-invasive replica technique is quicker in practice (10 minutes vs 27 minutes for each specimen) and, furthermore, the invasive technique requires an additional 24 hours for gypsum setting.

While digital radiography has also been used to evaluate crown margins, clinical evaluation is also required, and metal ceramic crown margins may produce false positive results and all-ceramic crown margins false negative results [16]. The introduction of different ceramic materials with various radiodensities into the market demands further research into the reliability of radiography for assessing crown margins [17,18]. Therefore, here we examined factors that might influence radiographic interpretation of crown margins to reach a consensus on the use of radiography for assessing the marginal fit of extra-coronal restorations. The null hypothesis was that PID angle and margin design have no effect on detecting an open crown margin.

## Materials and methods

The Research Ethics Committee of King Abdulaziz University approved this *in vitro* study (196-12-22).

**Fig 1:**
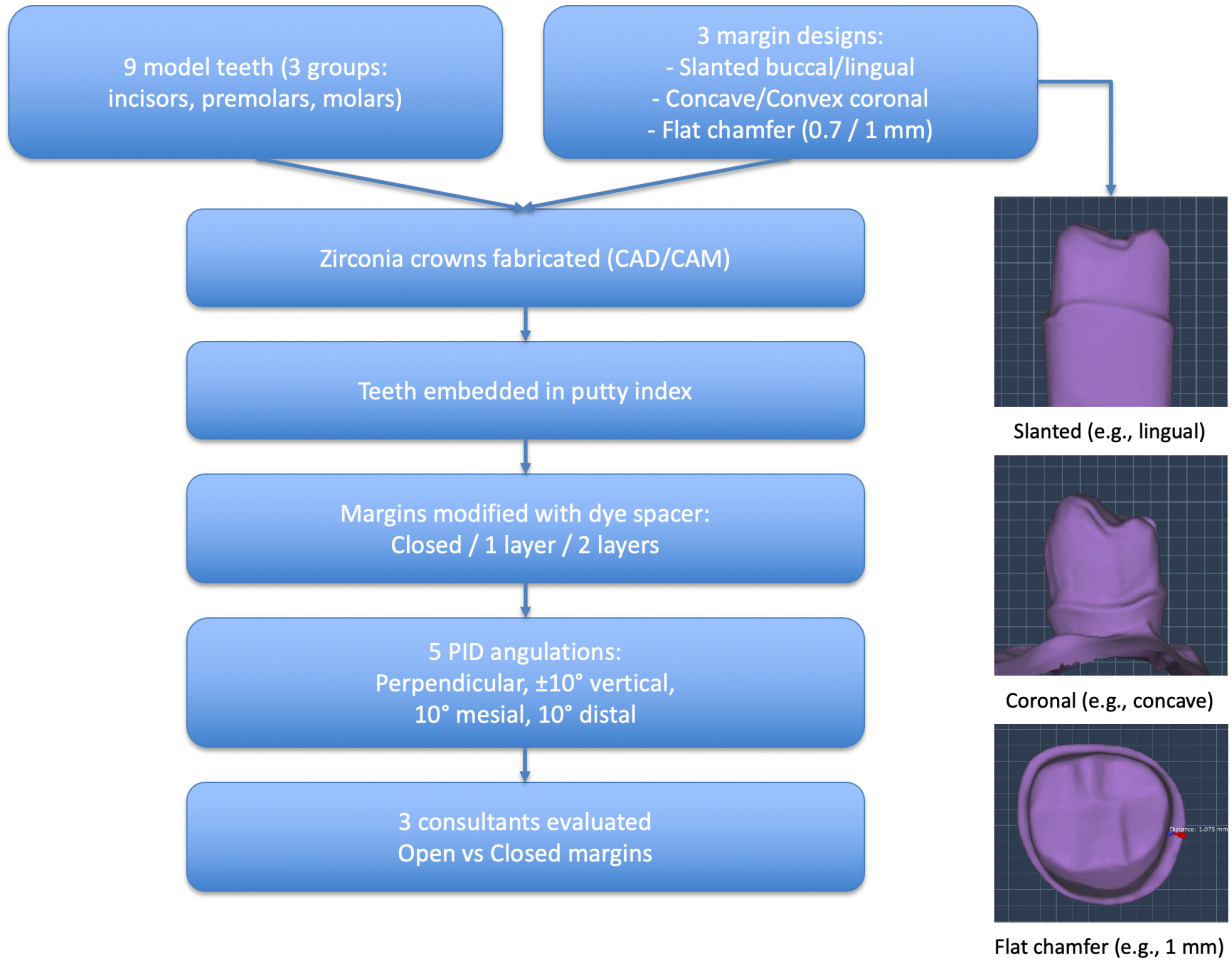
Overview of the study design.

An overview of the study design is shown in Fig 1. Nine plastic KaVo model teeth were divided into three groups (n = 3 per group): upper central incisors, upper premolars, and lower molars. Teeth were prepared by the consultant prosthodontist for zirconia crowns with three different finish line designs: in group 1, three teeth were prepared to have mesial margins slanting lingually and distal margins slanting buccally; in group 2, three teeth were prepared to have mesial margins concave coronally and distal margins convex coronally; and in group 3, three teeth were prepared to have mesial margins with a 0.7 mm-wide flat chamfer and distal margins with a 1 mm-wide flat chamfer (S1 Table).

Teeth were placed on the typodont, and a putty index was taken and cut in half for use as a verification guide to assess the reduction size: 2 mm occlusal/incisal reduction, 1 mm axial wall reduction, and 1 mm finish line width for groups 1 and 2 and 0.7/1 mm finish line width in group 3, with 10–20° total occlusal convergence, according to [19].

Round-end tapered diamond burs (1 mm thick) were used for axial wall and finish line preparations, barrel diamond burs for occlusal preparations, and American football burs for the palatal surfaces of the anterior teeth.

**Fig 2:**
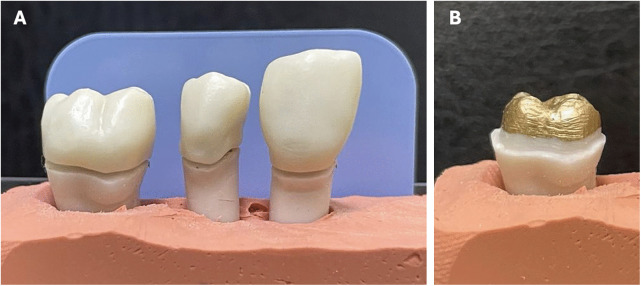
Preparation of model teeth. **(A)** Embedding of model teeth in a putty index for radiography. **(B)** Application of a layer of 14-micron dye spacer to the prepared teeth, maintaining a 1 mm distance from the margin.

Plastic teeth were considered as dies and were then scanned in the laboratory using a mes-iCore-I3DScan scanner (Eiterfeld, Germany) to mill nine different monolithic multilayer zirconia crowns. Teeth were then embedded in custom-made putty index to secure the teeth for radiographs. The finish line was 0.5 mm above the putty index to allow direct visual inspection with a sharp dental explorer. Each group of three teeth was embedded in one putty index with a space of 3–4 mm between teeth (Fig 2A).

A consultant radiologist used an intra-oral X-ray machine to take a set of five radiographs: the first set at baseline to verify a closed crown margin; the second set after application of one layer of 14 micron dye spacer to the prepared teeth maintaining a 1 mm distance from the margin to open the crown margin (Fig 2B), with the closed margin verified with a sharp dental explorer and radiographically, and a third set after application of two layers of 14 micron dye spacer, with the open margin verified with a sharp dental explorer and a subsequent set of radiographs. Layers of dye spacer were added to achieve an open crown margin both clinically and radiographically.

Radiograph 1 was taken by placing the PSP plate parallel to the long axis of the tooth and the X-ray beam perpendicular to both the plate and teeth; radiograph 2 with 10° of mesial shift; radiograph 3 with 10° of distal shift; radiograph 4 with +10° vertical angulation; and radiograph 5 with −10° vertical angulation. Radiographs were read and reported by a consultant radiologist and two consultant prosthodontists at each position-indicating device (PID) angle for both the mesial and distal surfaces at two time points 2 weeks apart. Open and closed margins were defined using a sharp dental explorer and confirmed with subsequent radiographs.

A post hoc power calculation using a bivariate normal model was performed using G*Power software. For α = 0.05, a sample size of 135 gave a power of 0.972. Data from the third set of open margin radiographs were imported into SPSS Statistics v20 (IBM Statistics, Armonk, NY). The chi-squared test was used to detect significant differences between groups. Binary logistic regression was used to determine predictors of open margins according to PID angles and margin design. Fleiss’ kappa was used to evaluate inter-rater reliability, while Cohen’s kappa was used to evaluate intra-rater reliability for categorical data and were interpreted as follows: < 0.001 poor, 0.001–0.20 slight, 0.21–0.40 fair, 0.41–0.60 moderate, 0.61–0.80 substantial, 0.81–1.00 almost perfect) [20]. A *p*-value <0.05 was considered statistically significant.

## Results

### Intra- and inter-examiner reliability of detecting open crown margins

The level of agreement between the 3 evaluators was almost perfect (Fleiss’ kappa 0.838). The level of agreement within each evaluator was moderate (Cohen’s kappa 0.451) for examiner one and substantial (Cohen’s kappa 0.664 and 0.802) for examiners two and three, respectively (S2 Table).

### Effect of different PID angles on open crown margins

Regardless of margin design, when the PID was placed perpendicular to the tooth, closed margins (i.e., false negatives) were detected almost as frequently as open margins (48.1% vs 51.9%). With a 10° mesial shift, 10° distal shift, and –10° vertical angulation, closed margins were reported in 35.2%, 42.6%, and 46.3% of cases, respectively. However, when X-rays were taken +10° vertical angulation, closed margins were detected 72.2% of the time. There was a statistically significant difference in the detection of open and closed margins between different PID angles (*p* = 0.002; Table 1).

**Table 1:** Detected open margins at different PID angles.

PID position indicator device (cone) angle	Closed margin	Open margin	Chi-square *p*-value
At right angle to the tooth	26 (48.1%)	28 (51.9%)	0.002
10 degrees mesial shift	19 (35.2%)	35 (64.8%)	
10 degrees distal shift	23 (42.6%)	31 (57.4%)	
(+) 10-degree vertical angulation	39 (72.2%)	15 (27.8%)	
(-) 10-degree vertical angulation	25 (46.3%)	29 (53.7%)	

### Effect of different PID angles and margin design on detected open crown margins

Regardless of the tooth, when margins were slanted lingually, there was a statistically significant difference in open/closed margin detection at different PID angles (*p* < 0.0001; Table 2), with closed margins mainly detected when the PID was at right angles to the tooth, 10° distal shift, and +10° vertical angulation. When margins were slanted buccally, there was a statistically significant difference in open/closed margin detection at different PID angles (*p* = 0.002), with closed margins mainly detected at right angles to the tooth and at +10° vertical angulation.

**Table 2:** Detection of open margins at different PID angles in different study groups.

Group 1	Mesial (slanted Lingual)			Distal (slanted buccal)		
PID position indicator device (cone) angle	Closed margin	Open margin	Chi-square *p*-value	Closed margin	Open margin	Chi-square *p*-value
At right angle to the tooth	7 (77.8%)	2 (22.2%)	0.000*	6 (66.7%)	3 (33.3%)	0.002*
10 degrees mesial shift	0 (0%)	9 (100%)		4 (44.4%)	5 (55.6%)	
10 degrees distal shift	6 (66.7%)	3 (33.3%)		0 (0%)	9 (100%)	
(+) 10-degree vertical angulation	9 (100%)	0 (0%)		8 (88.9%)	1 (11.1%)	
(-) 10-degree vertical angulation	3 (33.3%)	6 (66.7%)		3 (33.3%)	6 (66.7%)	
**Group 2**	**Mesial (concave coronal)**			**Distal (convex coronal)**		
**PID position indicator device (cone) angle**	**Closed margin**	**Open margin**	**Chi-square *p*-value**	**Closed margin**	**Open margin**	**Chi-square *p*-value**
At right angle to the tooth	2 (22.2%)	7 (77.8%)	0.558	8 (88.9%)	1 (11.1%)	0.394
10 degrees mesial shift	3 (33.3%)	6 (66.7%)		8 (88.9%)	1 (11.1%)	
10 degrees distal shift	3 (33.3%)	6 (66.7%)		5 (55.6%)	4 (44.4%)	
(+) 10-degree vertical angulation	5 (55.6%)	4 (44.4%)		7 (77.8%)	2 (22.2%)	
(-) 10-degree vertical angulation	2 (22.2%)	7 (77.8%)		6 (66.7%)	3 (33.3%)	
**Group 3**	**Mesial (flat chamfer 0.7 mm)**			**Distal (flat chamfer 1 mm)**		
**PID position indicator device (cone) angle**	**Closed margin**	**Open margin**	**Chi-square *p*-value**	**Closed margin**	**Open margin**	**Chi-square *p*-value**
At right angle to the tooth	2 (22.2%)	7 (77.8%)	0.006*	1 (11.1%)	8 (88.9%)	0.769
10 degrees mesial shift	2 (22.2%)	7 (77.8%)		2 (22.2%)	7 (77.8%)	
10 degrees distal shift	6 (66.7%)	3 (33.3%)		3 (33.3%)	6 (66.7%)	
(+) 10-degree vertical angulation	7 (77.8%)	2 (22.2%)		3 (33.3%)	6 (66.7%)	
(-) 10-degree vertical angulation	8 (88.9%)	1 (11.1%)		3 (33.3%)	6 (66.7%)	

When margin design was concave and convex coronally, there was no statistically significant difference in open/closed margin detection at different PID angles (*p* = 0.559 and *p* = 0.394, respectively). However, a convex coronal margin produced significantly more closed margins at all five PID angles.

With a flat chamfer of 0.7 mm, there was a significant difference in open/closed margin detection (*p* = 0.006), with 10° distal shift, + 10° vertical angulation, and –10° vertical angulation producing the most closed margin results. A finish line of 1 mm produced mostly open margins at different angulations with no statistically significant difference between open/closed margin detection (*p* = 0.769; Table 2).

### PID angle as a predictor of an open crown margin

The odds ratio of radiographic detection of an open 0.7 mm flat chamfer preparation when the PID was at right angles to the tooth and had a 10° mesial shift compared with when the PID was a −10° vertical angulation was 28 (95% CI 2.1–379.2; p = 0.012; S3 Table). Additionally, the odds ratio of radiographic detection of an open slanted buccal preparation when the PID was at +10° vertical angulation compared with −10° vertical angulation was 0.063 (95% CI 0.005–0.76; p = 0.03).

### Open/closed margin detection and margin design

There were no significant differences in open/closed margin detection between a slanted lingual and slanted buccal margin at any PID angulation (*p* = 0.399). However, there was a significant difference in open/closed margin detection between a concave coronal and a convex coronal margin and between a 0.7 mm and a 1 mm margin at any PID angulation (*p* < 0.0001 and *p* = 0.005, respectively; Table 3).

**Table 3:** Detected open margin in different margin designs.

	Group 1 (M-slanted lingual, D- slanted buccal)			Group 2 (M- concave coronal, D- convex coronal)			Group 3 (M- flat chamfer 0.7 mm, D- flat chamfer 1 mm)		
	Closed Margin	Open Margin	Chi-Square *p*-value	Closed Margin	Open Margin	Chi-Square	Closed Margin	Open Margin	Chi-Square *p*-value
Mesial	25 (55.6%)	20 (44.4%)	0.399	15 (33.3%)	30 (66.7%)	.000^*^	25 (55.6%)	20 (44.4%)	.005^*^
Distal	21 (46.7%)	24 (53.3%)		34 (75.6%)	11 (24.4%)		12 (26.7%)	33 (73.3%)	

### Margin design as predictor of open margins

The odds ratio of detection of an open crown margin if the margin design was concave coronal compared with convex coronal was 6.2 (95% CI 2.46–15.5; p < 0.001). Additionally, the odds ratio of detection of an open crown margin if the margin design was 0.7 mm rather than 1 mm was 0.29 (95% CI 0.12–0.70; p = 0.006) (S4 Table).

## Discussion

In this study, we investigated whether different PID angles and margin designs affect the detection of an open crown margin. Our analysis rejects the null hypothesis and suggests a significant effect of PID angle and margin design on detecting an open crown margin. Preparations were performed on anterior, premolar, and molar teeth and assessed at five different PID angles for six different margin designs. Radiographs were interpreted by one consultant radiologist and two consultant prosthodontists. Overall, closed margins (false negatives) were most commonly detected when the PID was at +10° vertical angulation (72.2% of the time), independent of the margin design. Closed margins were also commonly detected for slanted lingual and buccal margins when the PID was at right angles to the tooth or +10° vertical angulation, for concave coronal margins at +10° vertical angulation, and for convex coronal preparations at all angulations. A 1 mm chamfer, however, resulted in accurate open margin detection most of the time. Predictors of an open crown margin were PID angles at right angles to the tooth and 10° mesial shift when the margin was 0.7 mm. However, it was less likely that a slanted buccal open crown margin would be detected with +10° vertical angulation.

Our findings are consistent with Rice et al. [21], who reported significant marginal discrepancies according to different vertical X-ray angulations. Another older study used natural extracted teeth prepared to receive MOD casted onlays, with the mesial part receiving onlays with three sizes of open margin (0.01,0.05, and 0.1 mm), while a distal part acted as a closed margin control. The authors tested five vertical (+20°, + 10°, 0°, −10°, −20°) and five horizontal (distal 20°, distal 10°, 0°, mesial 10°, mesial 20°) angulations to detect open margins and concluded that the best angulation to detect an open crown margin is perpendicular to the long axis (0° in the horizontal and vertical planes) [8]. In that study, only a slight +10° to the vertical was deemed acceptable. Our results, however, suggest that a + 10° vertical angulation is not acceptable. Although the discrepancy in the results may be attributable to the different study designs and materials (onlay casting on natural teeth vs. full coverage zirconia crown and plastic teeth), we suggest using an angulation perpendicular to the long axis and to avoid +10° vertical angulation. Additionally, the authors did not account for different margin designs, while here we concluded a less favorable outcome with +10° vertical angulation in a slanted buccal preparation.

Another study compared conventional vs digital radiographic images (with and without additional applied filters) for detecting misfit in metal indirect restorations [22]. The size of the gap (0.2 mm gap vs 0.4 mm gap) and the type of restoration (inlay vs complete cast crown) had a significant impact on accuracy, and, although type of radiographic imaging (conventional vs digital) had no effect, the filter type did. The authors reported easier detection of marginal gaps with MOD inlays than complete crowns, and they recommended the use of either conventional radiography or original digital without enhancement to verify crown margins, especially for metal crowns.

Other studies have investigated the agreement between radiographic and visual inspection of a recurrent carious lesion under a restoration, reporting that unless a lesion is frankly present and active, it is unlikely to be detected radiographically [23,24]. In these studies, the authors questioned the complementary use of radiographs to detect recurrent caries under older restorations. Although not directly linked to our study, these studies still support the idea that radiographic interpretation and clinical imaging might not be aligned.

This study is limited by its use of plastic rather than natural teeth, which tend to be more radiopaque and might obscure gaps. To overcome this limitation, putty material was used to absorb some of the scattered radiation. Moreover, the size of the open margin was not measured, with an open margin defined using a sharp dental explorer and visualization. The open margin was achieved by placing two layers of dye spacer of 14 microns each; however, the exact marginal gap was not measured. Scanning electron microscopy or advanced technology like a KaVo scanner would be useful for verifying and standardizing the marginal gap.

The radiographic interpretation of the margins of zirconia crowns is therefore significantly influenced by PID angulation and margin design. The worst PID angle for detecting an open crown margin was + 10° vertical angulation, while the worst margin preparation was a convex coronal preparation design. A + 10° vertical angulation should be avoided when assessing adaptation of a zirconia crown margin, and a convex coronal finish line of less than 1 mm should be avoided as they are harder to detect radiographically when open. Further studies are now required to investigate the optimal radiographic angulation or setup for detection of open crown margins.

## Supporting information

S1 Table Finish line preparation design for each of the study groups. (DOCX)

S2 Table Inter- and intra-examiner reliability. (DOCX)

S3 Table Predictors of an open crown margin (binary logistic regression). (DOCX)

S4 Table Margin design as a predictor of an open margin (binary logistic regression). (DOCX)
