# Supplementary material for: Accurate radiographic interpretation of misfit milled zirconia crowns of different designs: An in vitro study
Source: PLoS One. 2026 Jan 8;21(1):e0338690. doi: 10.1371/journal.pone.0338690 (PMC12782363; doi:10.1371/journal.pone.0338690)
Supplement: S1 Table — (DOCX) [file pone.0338690.s001.docx]

**S1 Table. Finish Line Preparation Design for Each of the Study Groups**.

| Group | Teeth | Margin design |
| --- | --- | --- |
| 1 | 1 upper central incisor  1 upper premolar  1 lower molar | Mesial: slanted lingual  Distal: slanted buccal |
| 2 | 1 upper central incisor  1 upper premolar  1 lower molar | Mesial: concave coronal  Distal: convex coronal |
| 3 | 1 upper central incisor  1 upper premolar  1 lower molar | Mesial: 0.7 mm flat chamfer  Distal: 1 mm flat chamfer |
