# Supplementary material for: Accurate radiographic interpretation of misfit milled zirconia crowns of different designs: An in vitro study
Source: PLoS One. 2026 Jan 8;21(1):e0338690. doi: 10.1371/journal.pone.0338690 (PMC12782363; doi:10.1371/journal.pone.0338690)
Supplement: S2 Table — (DOCX) [file pone.0338690.s002.docx]

**S2 Table.** **Inter- and Intra-Examiner Reliability**.

|  | Fleiss' Kappa | Mean (st. dv.) | Interpretation |
| --- | --- | --- | --- |
| Inter-rater (Fleiss' Kappa) | 0.838 | 1.83 (1.278) | Almost perfect |

|  | Cohen's Kappa | p value | Interpretation |
| --- | --- | --- | --- |
| Intrarater (rater1) | 0.451 | 0.003 | Moderate |
| Intrarater (rater 2) | 0.644 | 0 | Substantial |
| Intrarater (rater 3) | 0.802 | 0 | Substantial |
