# Supplementary material for: Accurate radiographic interpretation of misfit milled zirconia crowns of different designs: An in vitro study
Source: PLoS One. 2026 Jan 8;21(1):e0338690. doi: 10.1371/journal.pone.0338690 (PMC12782363; doi:10.1371/journal.pone.0338690)
Supplement: S3 Table — (DOCX) [file pone.0338690.s003.docx]

**S3 Table.** **Predictors of an Open Crown Margin (Binary Logistic Regression)**.

| Margin Design | PID Angle | Sig. | Odds ratio | 95% C.I. for odds ratio | |
| --- | --- | --- | --- | --- | --- |
|  |  |  |  | Lower | Upper |
| Flat chamfer 0.7 mm | At right angle to the tooth | 0.012 | 28 | 2.07 | 379.2 |
| Flat chamfer 0.7 mm | 10 degrees mesial shift | 0.012 | 28 | 2.07 | 379.2 |
| Slanted buccal | (+) 10-degree vertical angulation | 0.03 | 0.063 | 0.005 | 0.76 |
