# Supplementary material for: Accurate radiographic interpretation of misfit milled zirconia crowns of different designs: An in vitro study
Source: PLoS One. 2026 Jan 8;21(1):e0338690. doi: 10.1371/journal.pone.0338690 (PMC12782363; doi:10.1371/journal.pone.0338690)
Supplement: S4 Table — (DOCX) [file pone.0338690.s004.docx]

**S4 Table.** **Margin Design as a Predictor of an Open Margin (Binary Logistic Regression)**.

|  | Sig. | Odds ratio | 95% C.I. for odds ratio | |
| --- | --- | --- | --- | --- |
|  |  |  | Lower | Upper |
| Concave coronal | 0 | 6.182 | 2.464 | 15.512 |
| 0.7 mm flat chamfer | 0.006 | 0.291 | 0.12 | 0.704 |
